# Breeding and migration performance metrics highlight challenges for White-naped Cranes

**DOI:** 10.1038/s41598-022-23108-w

**Published:** 2022-10-29

**Authors:** Batbayar Galtbalt, Tseveenmyadag Natsagdorj, Tuvshintugs Sukhbaatar, Claire Mirande, George Archibald, Nyambayar Batbayar, Marcel Klaassen

**Affiliations:** 1 Centre for Integrative Ecology, School of Life and Environmental Science, Deakin University, 75 Pigdons Road, Waurn Ponds, Victoria, 3216 Australia; 2 Wildlife Science and Conservation Center, Ulaanbaatar, Mongolia; 3 International Crane Foundation, Baraboo, WI USA

**Keywords:** Animal migration, Climate-change ecology, Conservation biology, Macroecology, Population dynamics, Wetlands ecology

## Abstract

Globally, habitat loss has been deemed a major threat to wetland bird populations. However, the underlying mechanism of population declines and variations in the birds’ vulnerability throughout their annual cycle is challenging to determine, yet critical for development of targeted conservation strategies. Over seven years, landscape water availability explained occupancy of breeding territories best when breeding performance, migratory performance, and annual survival of the White-naped Crane (*Grus vipio*) population in eastern Mongolia were studied. Also, the hatching success of eggs was positively correlated with water availability in addition to plant productivity. High ambient temperatures and large numbers of herder families (and hence more livestock) negatively affected hatching success. High water availability at Luan, a major stopover site increased migration speed during the cranes’ northbound migration to their breeding grounds. In contrast, when water conditions were favorable, the birds stayed longer at the stopover site during southbound migration. Increased human density reduced the use of the stopover site during northbound migration. Finally, cranes arrived early at the breeding grounds when ambient temperature was high in northeast Mongolia. Combining these findings with historical trends in key environmental factors on their breeding grounds explains the general decline observed in this population of cranes in recent decades. Extrapolating our findings with future climate predictions, the outlook seems poor unless urgent action is taken. Knowledge of the mechanisms underlying White-naped Crane population decline in eastern Mongolia identified in this paper should improve the effectiveness of these actions.

## Introduction

About one third of all global natural wetlands have disappeared over the last four decades due to climate change and other anthropogenic effects^1,2^. In East Asia environmental changes have included ongoing loss of wetlands accelerated by unprecedented socio-economic development^3,4^. These wetland degradations are thought to impact millions of waterbirds, causing population declines in both resident and migratory species^5–7^. Although studies have identified the associations between population declines and wetland degradation, the underlying mechanisms leading to these declines remain unclear. The three mechanisms most commonly proposed include reduced breeding performance (i.e., low recruitment due to low nesting, hatching, or fledging rates), reduced migratory performance (i.e., changes in departure and arrival dates, migration duration, number of stops), and low annual survival (i.e., juvenile and adult).

Decrease in breeding performance due to deterioration of nesting and foraging habitat at the breeding grounds is considered a primary cause of waterbird population declines^8,9^. Many waterbirds rely on aquatic plants as nest material and cover, without which nests become prone to predation^10^, trampling^11^, and extreme weather events^12^. Low food availability may result in reduced clutch size^13^ and nest attendance^14^, and increasing nest failure rates^15^.

For many waterbirds, migration is another essential part of the annual cycle that may be impacted by deteriorating environmental conditions^16^. Loss of wetlands is thought to reduce refuelling opportunities during waterbird migration^5,17^. Refuelling at spring staging sites is critically important for replenishing energy reserves as it can determine fecundity^18–20^. Many studies have shown that migratory birds adjust their migration in response to conditions en-route^21,22^, by either delaying or advancing their migration timing^23–26^ with potential carry over effects on their breeding performance^27^ and population dynamics^28^.

Finally, low survival can also lead to population declines^29^. Environmental change may not only impact recruitment but also directly or indirectly affect survival rates during both the breeding and non-breeding periods. For example, in some waterbird species positive correlations were documented between survival rates and precipitation^30^ as well as positive correlations between survival rate and ambient temperature at the non-breeding grounds^31^. While there are many more examples elsewhere^32–35^, no studies have addressed the impact of environmental conditions on the survival of waterbirds relying on freshwater ecosystems in East Asia. Building an understanding of the importance of environmental conditions (e.g., temperature, precipitation, water availability, vegetation, human activity) on waterbird breeding, migration and survival is crucial to disentangling the many potential mechanisms of decline. Such knowledge may also prove crucial in developing effective conservation management.

Cranes are the flagship avian family for wetlands with 13 of 15 species inhabiting these ecosystems, and with ten on the Red List of threatened species^36^. East Asia holds the highest crane diversity with eight migratory species, four of which are in decline. The White-naped Cranes (*Grus vipio*; hereafter WNC) native to East Asia is split into a western and an eastern population. The western population breeds predominantly in Mongolia and winters at Poyang Lake, China. The eastern population breeds in the Amur River basin on the border between Russia and China, and winters in South Korea and Japan. Despite these marked differences in distribution, there appears to be no genetic differentiation between the two populations^37^. Systematic annual counts from vantage points at the wintering sites indicate the western population declined by 83% between 1996 and 2014^38^ and the latest estimate at Poyang Lake from 2014 suggests that only 1,663 individuals remained^39^. A 60% decrease in territorial breeding pairs in the Ulz River basin in Northeast Mongolia was observed between 2000 and 2011^40^. Simultaneously, the eastern population of WNC appears stable and may be increasing^41^. Several factors might explain the increase of the eastern population. For instance, better habitat quality and artificial feeding at the wintering ground might have increased population fitness or immigration^37^. Despite these valuable insights we still lack a multi-year analysis allowing detailed understanding of the climatic and socio-economic drivers of WNC breeding performance, migration performance, and annual survival that may help explain the decline of the western population.

To understand the drivers and mechanisms of the population decline of WNCs, we investigated inter-annual variation in breeding performance (defined as territory occupancy, clutch size, and hatching success), migratory performance (defined as departure and arrival dates, migration duration, and number of stops), and annual survival (defined as adult and juvenile survival) in relation to ecological and socio-economic variables over a period of seven years. Next, we examined the historical pattern of the significant environmental drivers. With these analyses, we tested three hypotheses that (1) habitat degradation has disrupted WNC breeding performance resulting in low recruitment into the population, (2) habitat degradation has disrupted migratory performance, with potential delays or advances in arrival at the breeding grounds and corresponding effects on breeding performance, and (3) habitat degradation throughout the flyway has reduced the survival of WNCs resulting in population decline. In addition, we made qualitative predictions about the future of WNC based on two climate change scenarios of temperature and precipitation. Our study emphasizes that associations between breeding performance and its environmental drivers are the main mechanisms determining the decline of the western WNC population in East Asia and the contrasting population trends between the western and eastern WNC populations.

## Methods

### Determining the drivers of breeding performance

Every summer from 2013 to 2020 we monitored nests of WNCs in Khurkh Khuiten Nature Reserve in Northeast Mongolia, which is the core of western breeding distribution (48.2752°N, 110.3179°E). The reserve contains the Khurkh and Khuiten Rivers as well as a few other small tributaries of the Onon River, which is one of the main tributaries to the Amur River in Northeast Asia. Small-sized thermokarst lakes (i.e., lakes or ponds formed as the result of permafrosts thawing^42^) in the region form ideal breeding and staging wetland habitat for many waterbirds including cranes. Between 2014 and 2020, we annually estimated 53 to 71 pairs of WNC breeding within the reserve.

**Figure 1:**
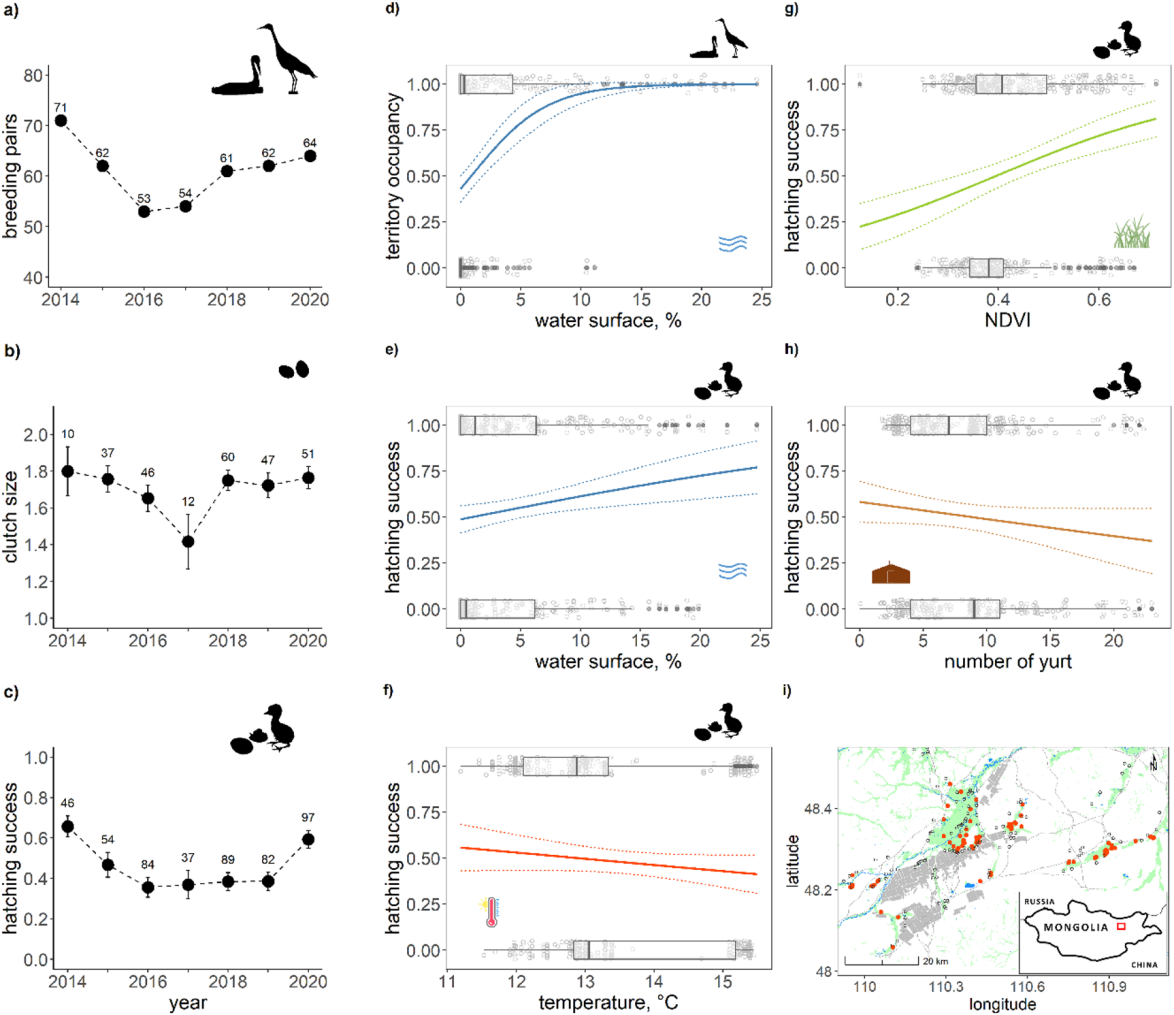
Breeding performance variation across the study period and significant explanatory variables for these variations in WNC. In panels (**a**–**c**) the total number of breeding pairs, average (± SE) clutch size and average (± SE) hatching success as a function of year are plotted with their respective sample sizes. In panels (**d**–**h**) significant relationships of these breeding performance indices with environmental factors are depicted (cf. Appendix Table A3), with distributions of explanatory variables presented as horizontal boxplots, including original data points (scattered grey dots) and outliers (black dots). In panel (**i**) a map of the study site is presented with wheat field in grey, wetlands in green and nest territories in red. All figures and maps were generated in RStudio v2022.02.2 (http://www.rstudio.com)^61^.

Every year, the monitoring involved three consecutive visits to all nesting territories in the reserve during early May to July. We used 20–60× telescopes to search an area of 1–3 km^2^ at a time, mapping all active nests in and around the lakes and wetland areas. To minimise disturbance, we visited nests only once and for a period of a maximum of ten minutes only. This typically occurred during the early incubation period in May. The nest monitoring enabled us to estimate breeding performance across years including territory occupancy (i.e., whether the pairs laid an egg in a known territory from the entire study), clutch size, and hatching success (i.e., whether an egg hatched). These three variables were then used for determining the annual variation of breeding performance and modelling response to environmental factors including plant productivity (Normalised Difference of Vegetation Index, NDVI, as proxy for plant productivity and invertebrate and small vertebrate food availability for cranes)^43^, water availability, ambient temperature, precipitation, number of herder families in the vicinity (proxy for impact of livestock and dogs; each herder family own at least 200 livestock and two dogs), and the distance to roads and wheat fields (proxies for other human activities).

We collated potential environmental factors for each nest location per year using different data sources. The mean NDVI and water availability (i.e., percentage of water surface) at each nesting location (within 500 m buffer) were obtained via Google Earth Engine. We used LANDSAT 8 top of atmospheric data for calculating the NDVI values following Dong et al.^44^. For water surface, we used JRC monthly water history v1.2 dataset^45^ since it is published and requires minimal pre-processing. The spatial resolution of NDVI and water surface are the same, as both use LANDSAT satellite images with a spatial resolution of 30 m. The temporal resolutions are every 16 days and monthly for the NDVI and water surface, respectively. The mean air temperature (i.e., 2 m above surface) and the total precipitation data within a 500 m buffer were extracted from the European Centre for Medium-range Weather Forecasts’ (known as ECMWF) ERA5-land monthly averaged data^46^, which was accessed via the Copernicus Climate Data Store. The spatial resolution of the ERA5 dataset is approximately 9 km. We collated these environmental datasets for May and June, the peak nesting period for WNCs. Finally, we estimated number of herder families or yurts within a five kilometre buffer from each nesting location, and the direct distance to the nearest road and wheat field using gBuffer, over and gDistance functions of R package rgeos^47^. The yurts, roads, and wheat fields were digitized from satellite images.

Once we collated above breeding performance measures and their associated environmental variables, we ran three logistic regression models using binomial Generalized Linear Models (GLM) to investigate the effects of the environmental factors on breeding performance of WNC. The GLM models included NDVI, water availability, mean ambient temperature and precipitation, number of yurts, and the distance to roads and wheat fields as the explanatory variables, and the territory occupancy (nested territory = 1, unnested territory = 0), clutch size (two eggs = 1, one egg = 0 to fit binomial GLM), and hatching success (hatched egg = 1, failed egg = 0) as the response variables in each model. All explanatory variables were scaled to enable the comparison between effect sizes. Prior to running the models, we examined the data for multicollinearity of explanatory variables using Variance Inflation Factor (VIF). Variables with a VIF > 3 were excluded from the analyses^48^. We also checked collinearity among explanatory variables and found that all correlation coefficients among the explanatory variables were |r|≤ 0.58, which is below the cut‐off value of 0.7^49^. For each GLM, we tested all combinations of the explanatory variables using the dredge function of the MuMln package^50^ to obtain model averaged parameter estimates. The top models were selected using the delta AIC criterion for which we used a threshold of two^51^.

### Determining the drivers of migratory performance

To determine the migration performance of WNCs, we deployed six Argos and 18 GPS-GSM transmitters on 24 individuals (11 juveniles and 13 adults) of WNCs at the northeast Mongolian (48.2752°N, 110.3179°E) and Daurian (50.3570°N, 115.7926°E) breeding grounds over the years 2013–2017. We caught young birds just before fledging. Molting and flightless adults were captured opportunistically. Transmitters were deployed externally on the birds’ back or leg (see deployment details in appendix Table A1). We used total of 52 complete migration events (31 southbound and 21 northbound) across 20 individuals in the analyses. The fixes with location error greater than 100 m were excluded. Using the timestamps of the location fixes we determined key migration phenology estimates including departure and arrival dates, migration duration, and number of stopover sites for each migration event. We considered fixes above 47° N to be on the breeding grounds and fixes below 29.3° N to be on the wintering grounds. To identify stopovers, we first re-sampled the location fixes with two hours of fixing interval using the amt package^52^ and then filtered them with step lengths (i.e., the distance between consecutive fixes) lower than 10 km and zero ground speed. These stationary location fixes were then clustered based on their proximity to each other using hierarchical clustering in r. To this end we used 50 km as a threshold value for identifying clusters. The clustering of fixes identified 88 location clusters outside the breeding and wintering grounds. Finally we only considered clusters where birds stayed a minimum of two days to identify 31 true stopover sites^53^.

We investigated the four phenology measures outlined above in relation to environmental variables to understand what could explain their annual variations. To this end, we ran a total of eight GLMs for the four response variables (i.e., departure and arrival dates, migration duration, and number of stops) during both northbound and southbound migration. Each response variable was tested against four explanatory variables. Here we used the same data sources as described for breeding performance. The explanatory variables included NDVI, water availability, ambient temperature, and precipitation at the respective sites (i.e., the breeding ground, wintering ground, and a major stopover) and timeframes, which were identified based on their migration phenology. We identified regions of interest for the respective sites, for which the explanatory variables were extracted, using the 100% minimum convex polygon of stationary fixes of all individuals. The mean of all explanatory variables for February and March from Poyang (wintering ground) and the mean of April data from northeast Mongolia (breeding ground) were used for assessing departure and arrival dates of northbound migration in relation to environmental conditions. The mean of explanatory environmental variables for September and October from northeast Mongolia and November data from Poyang were used for testing departure and arrival dates of southbound migration. For testing the migration duration and number of stops, we used the mean of March and April environmental data for the Luan River catchment (stopover) for northbound and mean of October and November data for southbound migration. This Luan River catchment (hereafter Luan) was previously recognised as the major stopover in a number of studies^53–55^. To test for potential anthropogenic impacts on migration, we included annual human population density estimates as an additional covariate for testing departure date, migration duration, and number of stopovers. We obtained total human population estimates for each region of interest from the worldPop online database with 1 km resolution^56^. Next, we calculated the number of people per square kilometre for all sites. We used human population density of Poyang and northeast Mongolia for the models of northbound and southbound departures, respectively. Human population density of Luan was used for models testing migration duration and number of stopovers. Explanatory variables were scaled to enable comparison between effect sizes. For each GLM, we tested all combinations of explanatory variables using the dredge function of MuMln package and obtained averaged estimates for all models. As with the analyses of breeding performance, variables with a VIF > 3 were excluded from the analyses.

### Determining the drivers of annual survival

For individual identification, we color marked 36 adult and 282 juvenile WNCs on the Mongolian breeding grounds between 2013 and 2019. We caught juveniles within days prior to fledging, while adults were caught opportunistically when molting (i.e., flightless) adults were encountered. Unique combinations of three leg color bands were attached to the right tibiotarsus of each individual. The process of banding took on average 10 min. We did not encounter any unusual behavior upon releasing the birds. To estimate annual survival, we used the total of 852 resightings of 219 color banded individuals of which 94% were registered on the Mongolian breeding grounds through December 2020. We estimated annual survival of adult and juvenile WNCs separately, using a Bayesian population analysis model adapted from Kéry and Schaub^57^ using WinBUGS14 within the R and Rstudio framework. Birds were considered juvenile and adult when they were resighted before or after two years since hatching, respectively. For all birds, hatching date was considered to be the 1st of May. While assuming a fixed age effect in annual survival (Φ) we assumed annual resighting rate (*p*) to be identical for both age classes. The model was run using 3 Markov chain Monte Carlo (MCMC) simulations using 10,000 iterations each. Initial parameters were chosen randomly for each MCMC simulation. The last 5,000 iterations of each chain and a thinning factor of 6 were used to describe the posterior distribution of each parameter (i.e., their median and 95% confidence interval). For the accuracy of Φ and *p* estimation $\hat{R}$ was used, with values of $\hat{R}$ close to 1 identifying good fits and only accepting a model where all $\hat{R}$<1.02.

Next, we related annual survival estimates of both age groups with temperature, precipitation, NDVI, water availability and human population density at the Mongolian breeding grounds, Luan, and Poyang to test whether these environmental variables could explain variation in annual survival. To this end, and to reduce the family-wise error rate through conducting a large number of tests, we identified principal components of these five environmental variables across the various regions and time points of interest using the FactoMineR package in R^58^. We used the same data sources and regions of interest as described in the previous sections. However, the time periods were different, using the averages of April to September to represent summer conditions on the Mongolian breeding grounds, and the averages of September to October and March to April to represent conditions during southbound and northbound migrations at the Luan stopover site. Lastly, we used the November to February data to represent wintering conditions at Poyang Lake. We used annual human population density estimates for each site. We were able to significantly reduce the total of 19 variables (i.e., four environmental variables for four seasons and annual human population density estimates for each site) to as few as six principal components explaining 100% of the variation (Appendix Fig. A1). We ran a GLM model for adults and juveniles separately, with annual survival as response variable and the first five principal components as the explanatory variables. For each GLM, we tested all combination of explanatory variables using the dredge function of MuMln package and obtained averaged estimates of all models.

### Determining the historical trends of key environmental factors

**Figure 2:**
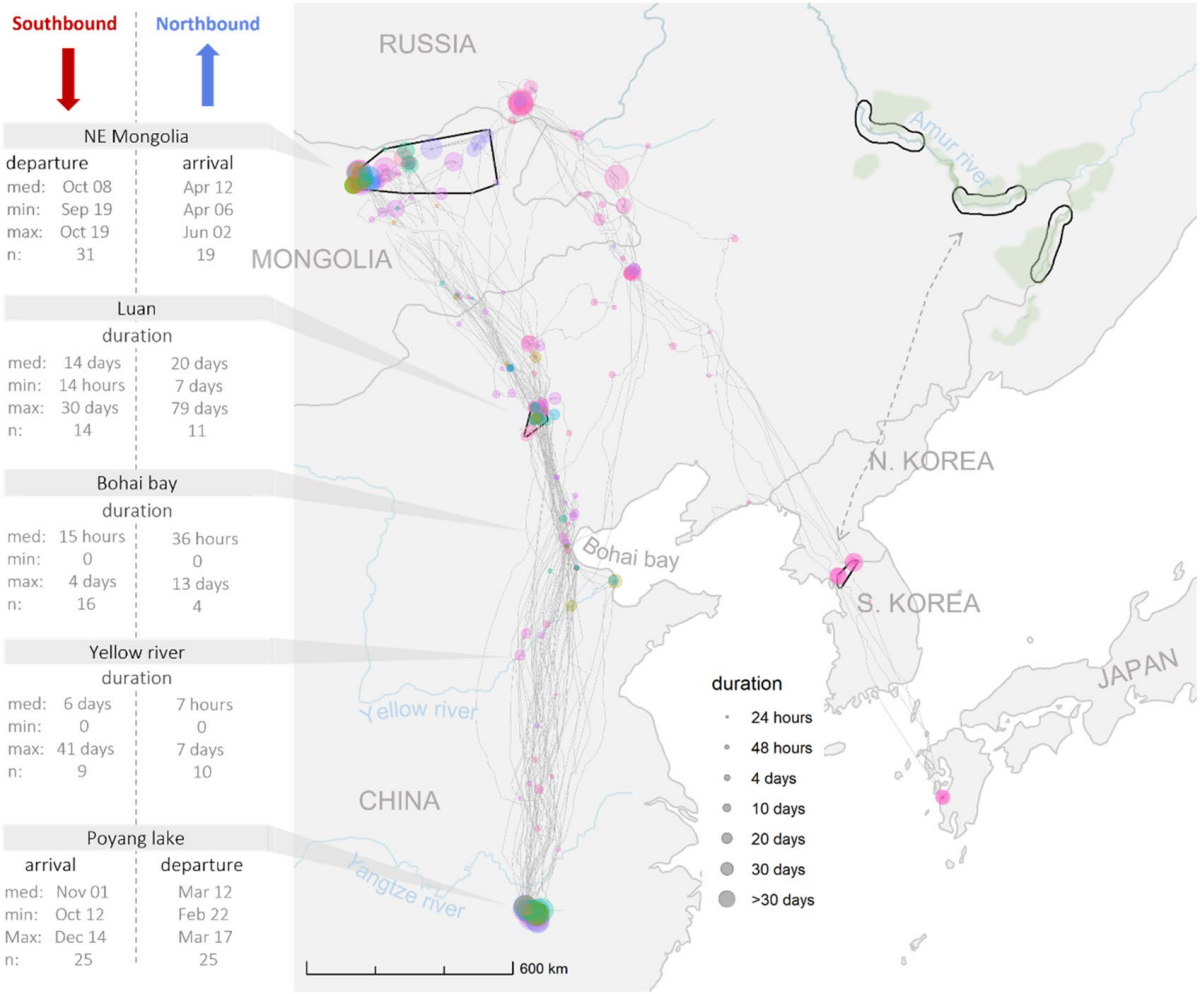
Migration routes (grey lines) and major stopovers (coloured circles) of WNC. The colours of the circles indicate different individuals, while each circle’s radius indicates the stopover duration. The migration routes of the eastern population are indicated with dashed lines based on Higuchi, Pierre^62^. The black polygons are the regions used for extracting environmental variables. The map was generated in RStudio v2022.02.2 (http://www.rstudio.com)^61^.

We explored historical patterns of key environmental factors, which the above analyses showed had a significant effect on breeding and migratory performance of WNCs, to identify if their patterns could explain the recent population decline. When exploring these key environmental factors, we also obtained the matching data for the eastern breeding grounds to allow a tentative comparison between the two breeding populations. For the eastern population, we used the distribution map in Mirande and Harris^41^ adding a 25 km buffer to each side of the three core areas they indicated along the Amur River basin (Fig. 2). We extracted the historical data for the regions of interest using the same data sources as described in previous sections, with the exception of NDVI, where we instead used LANDSAT 5 and 7 images. NDVI and water availabilities were extracted for years 1987–2020, and temperature and precipitation data for years 1950–2020. We obtained human population density estimates for years 2000–2020. In addition, we obtained data on annual livestock numbers at the Mongolian breeding grounds from the Mongolian statistical office for the years 2000–2020. When plotting these historical datasets, we standardised all factors except NDVI to allow comparison between sites. Water availability was standardised for each site by setting the maximum percentage of water surface ever reached in that site at 100%. For ambient temperature and precipitation, we took the long-term average as the reference point, from which we calculated the absolute anomalies. Lastly, for human population density, we took the estimates in the year 2000 as a reference and calculated proportional changes relative to this initial value. We used GAM smoother to evaluate trends over time in all environmental factors^59^.

### Ethics approval

All procedures performed during nest monitoring and catching, handling and tagging of birds complied with the relevant international guidelines and regulations^60^, and were approved by the ethics committee of the Ministry of Environment and Tourism in Mongolia (Licence: 04/2550).

## Results

### Breeding performance

The breeding performance of WNCs varied considerably among years with 10, 8, and 26% variation in number of breeding pairs, clutch size and hatching success, respectively (Fig. 1a–c). The clutch size stayed stable but showed a sudden drop in 2017. Hatching success was low (below 50%) during years 2015–2019, but higher than 50% in 2014 and 2020.

All explanatory variables were included in the top models of territory occupancy and hatching success, indicating the importance of habitat quality for breeding (Appendix Table A2). Water availability had a positive effect on territory occupancy and hatching success (Fig. 1d–e, Appendix Table A3). Eggs were also more likely to hatch when plant production (NDVI) was high and temperatures relatively low and there were fewer yurts in the vicinity of nesting sites (Fig. 1f–h). Clutch size was not affected by any of the environmental variables investigated.

### Migratory performance

All tracked individuals wintered at Poyang Lake in China. However, one individual from the Daurian breeding grounds altered its wintering site to the Demilitarized Zone (DMZ) of the Korean peninsula and Izumi in Japan after initial migration to Poyang Lake (Fig. 2). The analyses of migratory performance was limited to those birds migrating to Poyang Lake to focus on the western population for which we have adequate data.

**Figure 3:**
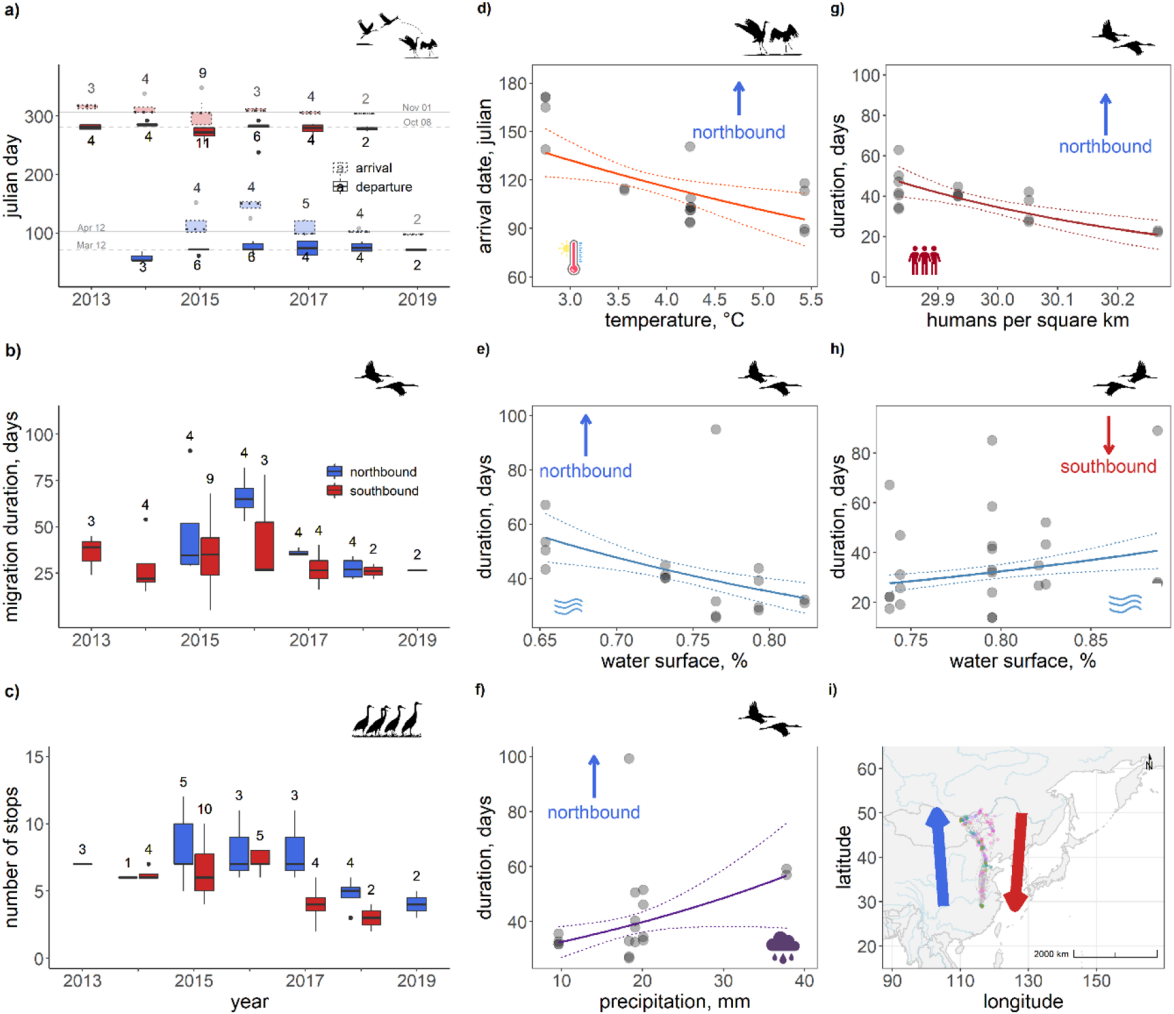
The migratory performance across the seven years of the study and their relationship with significant explanatory variables in WNCs. In panels (**a**–**c**), boxplots of arrival and departure dates, migration duration and number of stops as a function of year are plotted. The number of birds are indicated. Red and blue box plots refer to southbound and northbound migration respectively (cf panel **i**). In panels (**d**–**h**) significant relationships of these migratory performance indices with environmental factors are depicted (cf. Appendix Table A4), using the partial residuals of the response variables and depicting the marginal effect response curve for each relationship. The map and figures were generated in RStudio v2022.02.2 (http://www.rstudio.com)^61^.

WNCs took similar routes during southbound and northbound migrations. Birds departed their breeding sites from mid-September through mid-October heading southeast to their major stopover sites at the upper reaches of the Luan River in northern China, where 78% of individuals stayed on average 14 days. Next, they moved south to Bohai Bay (Beidangang or Yangcheng reservoirs near Tianjin) where some individuals spent a day while others continued moving further south. Then they moved on to the Yellow River basin where some individuals stopped for a few days before moving on to their main wintering sites at Poyang Lake. Birds arrived at their wintering sites from mid-October through mid-December. After spending the winter at Poyang Lake, birds started departing north again from late February until late March. Their first stop was at different parts of the Yellow River basin where birds stayed for a few hours to 3 days, while a few migrated directly to Bohai Bay for a stopover varying in time for a few hours to up to 13 days. Their next stop was at the upper reaches of the Luan River where they staged for an average of 20 days before heading north to the Mongolian breeding grounds. Birds arrived at the breeding grounds over a two-month period spanning early April until the first days of June (see summary in Fig. 2). Along the migration route of the western population, we found up to 31 stopover sites where cranes had stayed for more than two days. Five of these sites were clustered along the Luan River. WNCs tended to use a greater number of stopover sites during northbound compared to southbound migration. The duration of northbound migration was typically longer (median = 35 days, min = 22, max = 91, n = 18) than the duration of southbound migration (median = 29 days, min = 5, max = 78, n = 25). Variation in migration phenology across years showed that northbound migration in 2016 was particularly long (median = 65 days, min = 53, max = 82, n = 4) with the first individual arriving at the breeding grounds at least three weeks later than the first arrivals in other years. In that year cranes not only spent more time at stopovers during their northbound but also their southbound migration (Fig. 3).

The GLM models showed that environmental conditions had a noticeable effect on both northbound and southbound migration durations and on the timing of arrival at the breeding grounds (Appendix Table A4, Fig. 3). Northbound migration duration was shorter when human density and water availability were high, but high precipitation increased northbound migration duration. Higher ambient temperature advanced arrival date on the breeding grounds. In contrast, high water availability increased southbound migration duration indicating longer stays when stopover sites had more water.

### Annual survival

**Figure 4:**
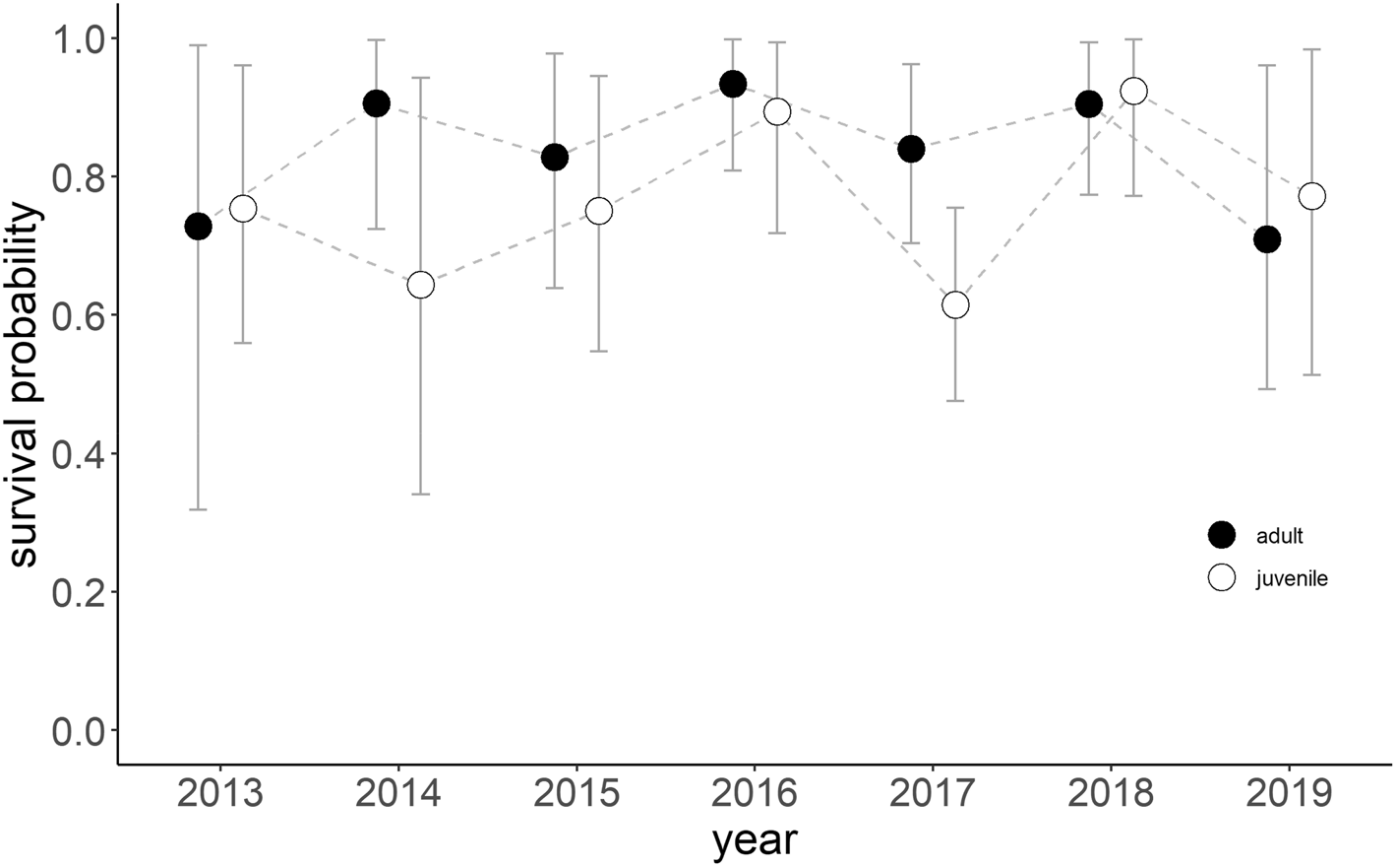
Annual survival probability of adult (black dot) and juvenile (white dot) WNC. The error bars indicate ± 95% confidence intervals. The figure was generated in RStudio v2022.02.2 (http://www.rstudio.com)^61^.

Over the seven years study period, resighting rate showed a steady increase ranging from 29% on average in the first year to 78% in the last year of study. Annual survival of WNCs varied little between years with 10 and 15% variation in annual survival of adults and juvenile, respectively (Fig. 4). On average, adult survival was 84%, reaching its lowest survival of 71% in 2019 and highest survival of 93% in 2016, although it should be noted that for all years the 95% confidence intervals of the annual survival estimates were overlapping. The average juvenile survival was 76% with lowest 61% in 2017 and highest 92% in 2018, but also here the 95% confidence intervals of the annual survival estimates were overlapping for all years. Possibly due to the lack of significant annual variation in survival rates in both age groups, no relations were found with annual variations in environmental conditions. The GLM models on annual survival in relation to five principal components comprised of 19 environmental variables showed none of the components had an effect on annual survival of adult and juvenile WNCs (Appendix Table A5).

### Historical trends of key environmental factors

**Figure 5:**
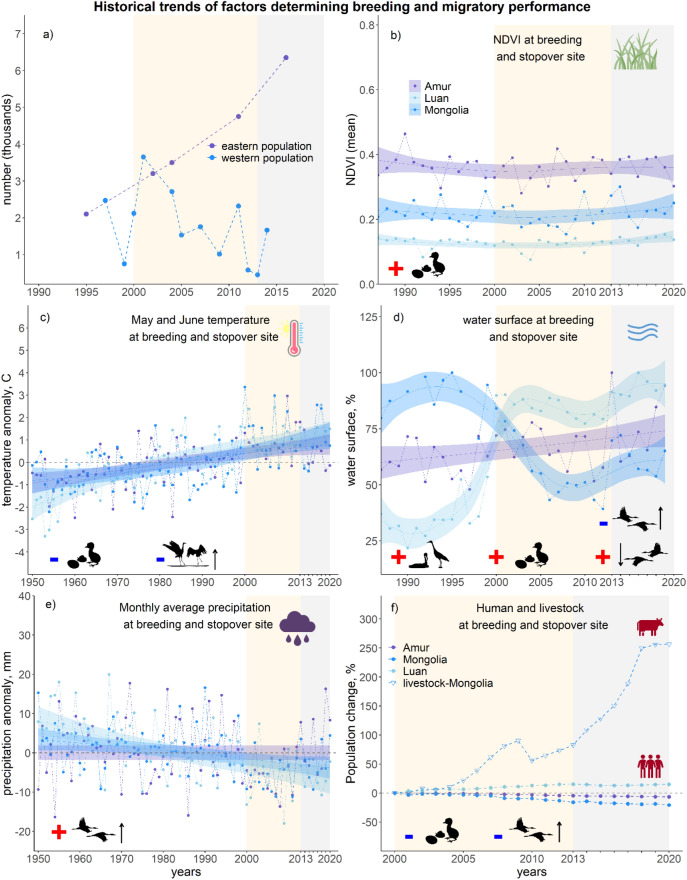
Historical trends of WNC populations and environmental factors potentially affecting breeding and migratory performance. Population estimates (panel-**a**) of western population were obtained from Wang, Fraser^39^, while estimates of eastern population were obtained from Wetland International’s database. Historical trends of NDVI (**b**), temperature (**c**), water availability (**d**), precipitation (**e**), annual changes in human population density and livestock for both western (blue) and eastern (navy) breeding grounds (**f**), and major migratory stopover site of the western population at Luan (light blue) are also depicted in panel (**b**–**f**). Grey shading indicates the timeframe for which nest monitoring was conducted. Note that the time axis may differ from panel to panel and that for ease of comparison, the yellow shading indicates a period of major drought at the breeding grounds of Mongolia and Amur. All figures were generated in RStudio v2022.02.2 (http://www.rstudio.com)^61^.

Although NDVI was a key factor positively affecting hatching success, it has been the most stable environmental factor over the last three decades, not showing any major temporal trend (Fig. 5b). Also noteworthy is the relatively high mean NDVI at the breeding grounds of the eastern population in the Amur region compared to Mongolian breeding grounds.

The ambient temperature, negatively affecting hatching success and northbound arrival date, has been the only factor for which the overall trend shows consistent increase for all sites since 1950 (Fig. 5c). The rate of increase was slightly higher at the Mongolian breeding grounds of the western population compared to breeding grounds of the eastern population in the Amur region. Both breeding grounds experienced above average temperatures during their breeding season since year 2000.

Water availability, positively correlated with territory occupancy and hatching success, and negatively correlated with northbound migration duration and positively correlated with southward migration, has been highly dynamic over the past three decades, showing contrasting site-specific patterns (Fig. 5d). The Mongolian breeding grounds of the western population have seen the greatest changes with high water availability until 1995, decreasing sharply to only half the water surface area by 2004. Although fluctuating since, with above average precipitation in 2013 and 2019, this situation has by and large remained unchanged. While the breeding grounds in Amur have seen higher inter-annual fluctuations in water availability, the trend is positive. The major migratory stopover site at Luan has seen a positive trend in water availability over the past three decades.

Precipitation, positively correlated with northbound migration duration, decreased at the Mongolian breeding grounds, while it showed a stable long-term trend at the breeding grounds in the Amur region (Fig. 5e). However, both breeding grounds have experienced overall below average precipitation during the first decade of this century, with a tendency to return to overall average levels notably in Amur River basin since 2013. The major migratory stopover site at Luan has seen a negative trend in precipitation over the past seven decades.

Lastly, we had found that the number of yurts as a proxy for number of people and livestock (Fig. 1h), negatively correlated with hatching success. While number of people has slightly decreased at both breeding grounds in recent times, the number of livestock has increased two-fold at the Mongolian breeding grounds since year 2000 (Fig. 5f). The human population density at the Luan stopover, negatively correlated with northbound migration duration, slightly increased over the same period (Fig. 5f).

## Discussion

The results of the current study indicate that water availability and plant productivity (NDVI) are of key importance in explaining annual variations in breeding and migratory performance of WNCs, followed by human activity, temperature, and precipitation. The negative effects of temperature on arrival date at the breeding grounds, in combination with 70-years of rising temperatures, promote a trend for earlier arrival. Although in principle the availability of the breeding grounds for reproduction is thus gradually being extended, the historic trends of reduced water availability and increasing temperature and human activity might have played a key role for population decline of WNC, with reduced nesting attempts, poor hatching success and low adult survival. Reputedly, birds are under less time stress during southbound migration and WNCs apparently make use of favourable foraging conditions during these travels, migration duration being positively affected by water availability in autumn. A major change along the WNC’s flyway was the establishment of a dam across the upper Luan River, resulting in a major increase in water availability since 2000. Annual survival appeared unaffected by any of the environmental variables investigated. However, we should note that we did not link environmental conditions directly to the survival and future studies should address this gap when additional data becomes available.

Taken together and considering that our conclusions are based on a limited time frame of seven years and can only be considered tentative, our findings primarily support hypothesis i (habitat degradation has disrupted breeding performance) in explaining population declines in recent years. Hypothesis ii (habitat degradation has changed migratory performance) and hypothesis iii (habitat degradation throughout the flyway has decreased survival) are unsupported by results of current study. Climate change is projected to intensify with increasing temperature and more erratic precipitation on the western breeding grounds including eastern Mongolia (Appendix Fig. A2). These climatic processes and gradually increasing human activity on the breeding grounds, in combination with the negative effects of temperature and human activity on breeding performance, sketch a bleak picture for WNCs. Notably, if these increased temperatures are ultimately also leading to desiccation and reducing availability of breeding habitats, given that we have demonstrated that both NDVI and water availability have a major positive effect on breeding performance of WNCs.

### Habitat degradation disrupts breeding performance

In decreasing order of importance, we found that water availability, plant productivity, human activity, and temperature affect annual variations in breeding performance of WNCs. Wetlands with sufficient water and reedbeds are essential for waterbird breeding, providing both nest protection and abundance in food resources^63^. Our results showed water availability was the only influential factor of the seven variables studied for WNC territory occupancy and also contributed significantly to the hatching success of the eggs. However, plant productivity, which is promoted by water availability in spring^64^, had the strongest effect on hatching success. In line with this findings, Bradter, Gombobaatar^65^ found that WNC nests with good reed coverage had better hatching success. These findings point towards the opportunity for good nest concealment to be of prime importance for successful breeding. In addition, as plant productivity is possibly also a good proxy for food availability^43^, the increase in foraging efficiency (i.e., more energy intake) of adults prior and after laying may lead to better reproductive output. The negative effect of human activity (yurts) in the vicinity of WNC nests on hatching success might imply two potential threats: nest trampling by livestock and predation by domestic dogs. Although we were not able to disentangle their respective effects directly, opportunistically placed camera traps at ten nesting territories revealed over 95% of images taken were triggered by livestock, with several incidences of nest trampling by horses and predation by dogs, suggesting a high rate of disturbance and loss of nests to these causes (Tseveenmyadag, personal comm. 2021). Also other studies suggest negative effects of human activities on WNC territory occupancy and adult vigilance^40,66^. Finally, hatching success decreased with an increase in ambient temperature. However, this effect may again be more related to water availability rather than temperature directly, given that ambient temperature is negatively correlated with precipitation. The historical trends in all the key environmental variables are indicative of ongoing wetland degradation at the western breeding grounds over the past two decades, with a notable decrease in water availability, increased temperatures, and decreased precipitation (Fig. 5). In addition, increasing livestock density (e.g., extensive pastoralism) may have additionally contributed to this degradation^67^. Viewing the changes in breeding performance of WNCs along with the climatic and socio-economic changes on the western breeding grounds suggest that increasingly degrading wetlands explain the decline in this population. Therefore, it is essential to monitor the breeding population closely and further improve these analyses by incorporating other performance metrics such as fledging success and recruitment rates.

### Cranes adjust arrival date at the breeding grounds

We found that temperature, precipitation, water availability, and human density affected annual variations in migratory performance of WNCs. Notably, northbound migration was faster in years with high water availability and human density at their main, Luan, stopover site. Also, cranes typically arrived earlier in years with warmer ambient temperature at the breeding grounds. Northbound migration was slow when there was more precipitation, which likely fell in the form of snow in that period of the year. During southbound migration, cranes spent more time at stopovers when water availability was high. In general, spring migration is an important life-history event, with timely arrivals allowing for the successful establishment of nesting territories and breeding^16^. To facilitate a timely arrival, conditions at stopover sites progressively closer to the breeding grounds may enable predicting environmental conditions at the breeding grounds^68^. Every year, despite departing on similar dates from the wintering grounds, the arrival dates at the breeding grounds varied considerably between years, with major delays in 2016, suggesting tuning of stopover lengths to wait for better conditions at the breeding grounds. Due to the limited sample size, we were not able to confidently test if variable arrival dates influenced breeding performance. Yet, with the data available thus far, we found no effect of laying dates on hatching success (Appendix Table A7). In short, while migratory performance of WNC is influenced by environmental factors such as temperature, precipitation and water availability, these factors and changes therein in recent times are unlikely to explain the recent population decline.

### Annual survival could explain population decline

The gradually increasing expansion of the anthropogenic landscape in their flyways is thought to negatively affect many waterbird species by limiting suitable habitat availability^6^ and increasing disturbance at their stopovers^69^ with potential consequences for their survival^70^. Also WNCs are considered to be suffering from habitat degradation along their migratory route^55^ and at their breeding grounds (this study), we could not show this to have an effect on survival using the seven years of annual survival data available to date; there was no clear pattern in annual survival rates, and it did not relate to any of the environmental variables that we tested. However, comparing the stable adult annual survival rate (84% in this study) of WNCs over the seven year study period with related species, it appeared lower than in Whooping Crane (*Grus americana*) (94.4%)^71^ and Sandhill Crane (*Grus canadensis*) (94.2%)^72^ in North America, of which populations are both increasing^71–73^. Studies on wild Whooping and Sandhill Cranes have shown that the populations of these species are most sensitive to survival rates of breeding adults^71,72^. For example, a reintroduced Whooping Crane population began to decline with under 90% adults survival^74^. Therefore, further investigation is needed to evaluate how this relatively low annual survival rate of WNCs is determining the population decline in combination with recruitment rates. Causes and locations of adult mortality need to be better understood and effective actions should be taken to reduce mortality. Our data did not allow us to assess and identify bottlenecks in the survival of WNC during different stages of their annual cycle (i.e., nonbreeding vs. breeding birds). However, with resighting rates being steadily on the increase, we are hopeful to be able to obtain more precise and temporally detailed survival estimates in the future.

### The future trends and conservation implication

Our seven-year study identified a number of potentially critical drivers of WNC population dynamics and through extrapolation as well as clarified historic population declines it has enabled us to make future predictions. Climate change is projected to intensify in the coming years, with increasing temperature and more erratic precipitation on the western breeding grounds of WNCs (Appendix Fig. A2, Fig. A3). These climatic processes in combination with increasing human activity are expected to result in further degradation of wetlands in the region. Over the past 50 years, many of the region’s wetlands have been converted to agricultural land^75–77^, reducing breeding habitat but also opening up alternative foraging opportunities for WNCs^53,78^. The use of these fields by cranes is causing human-wildlife conflicts due to real or perceived crop damage in many places^26,77,79–83^. The extent of this conflict for WNCs currently seems manageable, due to their low population size and the generally positive attitude of human communities towards cranes in East Asia. Feeding in agricultural lands also exposes cranes to accidental poison from ingesting seed treated with chemicals used to reduce loss to invertebrate pests. One of our tracked WNCs was involved in an incident where six WNCs were rescued after ingesting poison. Cranes are also at risk of ingesting agrochemicals used in bait for illegal harvest of other birds^41^. The WNC population wintering in Japan has concentrated at one artificial feeding site at Izumi^84^. However, this feeding site, attracting many other species, might increase the risk of disease outbreaks, such as of high pathogenic avian influenza^85^, which increasingly circulates in the region and elsewhere in the northern hemisphere, affecting wild birds and poultry^86^. Given the many global change threats potentially affecting WNCs and other wetland-dependent birds, it is crucially important to closely monitor and study their populations and implement appropriate management actions where problems are being detected. Since WNC breeding, wintering, and staging grounds stretch across a huge area and many countries, this can only be achieved through collaborative research efforts. Many of the WNCs key sites are unprotected^53–55^ including breeding sites, which our study indicates have decreased in suitability and may be key in explaining the species’ population decline. Though we did not link habitat conditions directly to survival in this study, conditions were clearly associated with breeding performance and therefore likely long-term productivity. Priority conservation actions could include (1) limiting human impacts at key sites to avoid disturbance and habitat degradation, notably during the breeding season, and (2) developing standard protocols to annually estimate population size (counts), recruitment (assess family size at staging and wintering grounds), and survival (colour marking and resighting of marked individuals) throughout the flyway to monitor the populations’ status and evaluate the effectiveness of mitigation measures.

## Supplementary Information

Supplementary Information.

## Data Availability

The dataset and R script used in this article has been deposited on Dryad (https://doi.org/10.5061/dryad.w9ghx3fqs).
