# Supplementary material for: Breeding and migration performance metrics highlight challenges for White-naped Cranes
Source: Sci Rep. 2022 Oct 29;12:18261. doi: 10.1038/s41598-022-23108-w (PMC9617902; doi:10.1038/s41598-022-23108-w)
Supplement: Supplementary file 1 — Supplementary Information. [file 41598_2022_23108_MOESM1_ESM.docx]

APPENDIX

**BREEDING AND MIGRATION PERFORMANCE METRICS HIGHLIGHT CHALLENGES FOR WHITE-NAPED CRANES**

*Batbayar Galtbalt, Tseveenmyadag Natsagdorj, Tuvshintugs Sukhbaatar, Claire Mirande, George Archibald, Nyambayar Batbayar, Marcel Klaassen*

Table A1. A summary of transmitter deployment.

| **tag ID** | **age at capture** | **date caught** | **cap_site** | **tag manufacturer** | **type** | **tag weight** | **system** | **interval** |
| --- | --- | --- | --- | --- | --- | --- | --- | --- |
| X130949 | ad | 12-Aug-2013 | Kharaat, Khurkh | NorthStar | leg mount | 45gr | Argos | 8 h |
| X130948 | Juv | 14-Aug-2013 | Bayanburd, Ovoot tolgoi | NorthStar | leg mount | 45gr | Argos | 8 h |
| X130950 | Juv | 14-Aug-2013 | Bayanburd | NorthStar | leg mount | 45gr | Argos | 8 h |
| 52409530 | ad | 19-Aug-2013 | Uvur Burd | CTT | leg mount | 60gr | GSM | 30 m |
| 27230893 | Juv | 9-Aug-2014 | Chukh lake | CTT | leg mount | 60gr | GSM | 30 m |
| X139082 | ad | 11-Aug-2014 | Khuiten, Khar lake | NorthStar | leg mount | 45gr | Argos | 8 h |
| 27231081 | Juv | 11-Aug-2014 | Khuiten, Ulziit | CTT | leg mount | 60gr | GSM | 30 m |
| X139083 | ad | 12-Aug-2014 | Duut | NorthStar | leg mount | 45gr | Argos | 8 h |
| 27233186 | ad | 14-Aug-2014 | Khuiten, Ulziit | CTT | leg mount | 60gr | GSM | 15 m |
| mwb1501 | Juv | 3-Aug-2015 | Kholboo shar | KoEco | back pack | 45gr | GSM | 2 h |
| mwb1502 | ad | 3-Aug-2015 | Kholboo shar | KoEco | back pack | 45gr | GSM | 2 h |
| mwb1503 | ad | 3-Aug-2015 | Kholboo shar | KoEco | back pack | 45gr | GSM | 2 h |
| mwb1504 | ad | 3-Aug-2015 | Kholboo shar | KoEco | back pack | 45gr | GSM | 2 h |
| 27233947 | Juv | 8-Aug-2015 | Borzya river, Russia | CTT | leg mount | 60gr | GSM | 15 m |
| X150758 | Juv | 9-Aug-2015 | Borzya river, Russia | NorthStar | leg mount | 45gr | Argos | 8h |
| 27234135 | Juv | 10-Aug-2015 | Borzya river, Russia | CTT | leg mount | 60gr | GSM | 15 m |
| ke1601 | ad | 17-Jul-2016 | Khonkhor lake | KoEco | back pack | 45gr | GSM | 2 h |
| nsm1606 | Juv | 14-Aug-2016 | Uvur Burd | KoEco | back pack | 45gr | GSM | 2 h |
| nsm1605 | ad | 18-Aug-2016 | Barkh, Dund Nomgon | KoEco | back pack | 45gr | GSM | 2 h |
| ke1604 | ad | 18-Aug-2016 | Barkh | KoEco | back pack | 45gr | GSM | 2 h |
| ke1606 | Juv | 20-Aug-2016 | Duut | KoEco | back pack | 45gr | GSM | 2 h |
| ke1607 | Juv | 20-Aug-2016 | Uvur Burd | KoEco | back pack | 45gr | GSM | 2 h |
| Rcees17111 | ad | 5-Aug-2017 | Binder Ovoo lake | KoEco | back pack | 45gr | GSM | 2 h |
| Rcees 17110 | ad | 10-Aug-2017 | Daltiin Uzuur, Norovlin | KoEco | back pack | 45gr | GSM | 2 h |

Table A2. Top models testing the effects of distance to wheat field (1), distance to road (2), NDVI (3), precipitation (4), temperature (5), water availability (6), and number of yurts within five kilometre of each nesting site (7) on nest occupancy, clutch size, and hatching success.

| Models | df | loglik | aicc | delta | weight |
| --- | --- | --- | --- | --- | --- |
|  |  |  |  |  |  |
| nest occupancy ~ 3 + 4 + 5 + 6 + 7 | 6 | -278.32 | 568.83 | 0 | 0.11 |
| nest occupancy ~ 1 + 3 + 4 + 5 + 6 + 7 | 7 | -277.48 | 569.21 | 0.38 | 0.09 |
| nest occupancy ~ 4 + 6 + 7 | 4 | -280.9 | 569.88 | 1.05 | 0.06 |
| nest occupancy ~ 5 + 6 + 7 | 4 | -280.98 | 570.05 | 1.22 | 0.06 |
| nest occupancy ~ 4 + 5 + 6 + 7 | 5 | -280.06 | 570.25 | 1.42 | 0.05 |
| nest occupancy ~ 6 + 7 | 3 | -282.27 | 570.59 | 1.76 | 0.04 |
| nest occupancy ~ 2 + 3 + 4 + 5 + 6 + 7 | 7 | -278.19 | 570.63 | 1.8 | 0.04 |
| nest occupancy ~ 1 + 4 + 6 + 7 | 5 | -280.32 | 570.77 | 1.94 | 0.04 |
| nest occupancy ~ 3 + 5 + 6 + 7 | 5 | -280.32 | 570.77 | 1.94 | 0.04 |
| nest occupancy ~ 1 + 5 + 6 + 7 | 5 | -280.34 | 570.81 | 1.98 | 0.04 |
|  |  |  |  |  |  |
| clutch size ~ 7 | 2 | -163.38 | 330.81 | 0 | 0.06 |
| clutch size ~ 6 + 7 | 3 | -162.75 | 331.59 | 0.78 | 0.04 |
| clutch size ~ 6 | 2 | -163.88 | 331.81 | 1 | 0.03 |
| clutch size ~ 4 + 7 | 3 | -162.99 | 332.07 | 1.26 | 0.03 |
| clutch size ~ 3 + 7 | 3 | -163.02 | 332.14 | 1.33 | 0.03 |
| clutch size ~ 1 | 2 | -164.1 | 332.25 | 1.44 | 0.03 |
| clutch size ~ 5 + 7 | 3 | -163.13 | 332.35 | 1.54 | 0.03 |
| clutch size ~ 2 + 7 | 3 | -163.33 | 332.75 | 1.95 | 0.02 |
| clutch size ~ 1 + 7 | 3 | -163.36 | 332.8 | 1.99 | 0.02 |
| clutch size ~ 5 | 2 | -164.38 | 332.8 | 1.99 | 0.02 |
|  |  |  |  |  |  |
| hatching success ~ 2 + 3 + 5 + 6 + 7 | 6 | -387.82 | 787.78 | 0 | 0.24 |
| hatching success ~ 1 + 2 + 3 + 5 + 6 + 7 | 7 | -387.52 | 789.23 | 1.46 | 0.12 |
| hatching success ~ 3 + 5 + 6 + 7 | 5 | -389.61 | 789.32 | 1.54 | 0.11 |
| hatching success ~ 2 + 3 + 4 + 5 + 6 + 7 | 7 | -387.77 | 789.74 | 1.96 | 0.09 |
|  |  |  |  |  |  |

*Table A3. Summary of models examining the effects of environmental variables on nest occupancy, clutch size, and hatching success as modelled using binomial Generalized Linear Models (GLM). Significant explanatory variables (p<0.05) are emboldened.*

| dependent variables | explanatory variables | slope | std. error | 95% CI | z | p-value |
| --- | --- | --- | --- | --- | --- | --- |
|  |  |  |  |  |  |  |
| nest occupancy per territory | (intercept) | 0.41 | 0.13 | 0.16/0.65 | 3.23 | <0.01 |
|  | NDVI | 0.16 | 0.14 | -0.10/0.43 | 1.20 | 0.23 |
|  | **water availability** | 1.41 | 0.26 | 0.90/1.93 | 5.40 | **<0.001** |
|  | yurts within 5 km | 0.20 | 0.12 | -0.03/0.43 | 1.74 | 0.08 |
|  | Temperature | -0.23 | 0.14 | -0.49/0.04 | 1.65 | 0.10 |
|  | distance to road | 0.06 | 0.1 | -0.14/0.26 | 0.61 | 0.54 |
|  | distance to cropland | 0.13 | 0.11 | -0.09/0.36 | 1.16 | 0.25 |
|  | Precipitation | -0.22 | 0.18 | -0.57/0.14 | 1.21 | 0.23 |
|  |  |  |  |  |  |  |
| clutch size | (intercept) | 0.91 | 0.13 | 0.65/1.18 | 6.75 | <0.001 |
|  | NDVI | 0.07 | 0.15 | -0.23/0.37 | 0.46 | 0.64 |
|  | water availability | -0.14 | 0.13 | -0.41/0.12 | 1.07 | 0.28 |
|  | yurts within 5 km | -0.21 | 0.14 | -0.49/0.06 | 1.52 | 0.13 |
|  | Temperature | -0.08 | 0.14 | -0.35/0.19 | 0.57 | 0.57 |
|  | distance to road | 0.03 | 0.14 | -0.25/0.30 | 0.19 | 0.85 |
|  | distance to cropland | 0.08 | 0.16 | -0.24/0.40 | 0.49 | 0.62 |
|  | Precipitation | 0.09 | 0.15 | -0.21/0.39 | 0.58 | 0.57 |
|  |  |  |  |  |  |  |
| hatching success | (intercept) | 0.28 | 0.09 | 0.11/0.44 | 3.21 | <0.01 |
|  | **NDVI** | 0.45 | 0.11 | 0.24/0.67 | 4.15 | **<0.001** |
|  | **water availability** | 0.3 | 0.1 | 0.11/0.49 | 3.12 | **<0.01** |
|  | **yurts within 5 km** | -0.23 | 0.09 | -0.41/-0.04 | 2.4 | **<0.05** |
|  | **temperature** | -0.22 | 0.1 | -0.41/-0.03 | 2.27 | **<0.05** |
|  | distance to road | 0.17 | 0.09 | -0.01/0.34 | 1.86 | 0.06 |
|  | distance to cropland | 0.09 | 0.11 | -0.12/0.30 | 0.84 | 0.4 |
|  | precipitation | -0.02 | 0.14 | -0.29/0.26 | 0.12 | 0.9 |
|  |  |  |  |  |  |  |

Table A4. A summary table of model averaged estimates of GLMs examining the effects of NDVI, water availability, temperature, and precipitation on the departure and arrival dates, migration duration, and number of stops during northbound and southbound migration. We used poisson distribution when testing the (scaled) effects of each variable.

| dependent variables | explanatory variables | slope | std. error | 95% CI | *z* | p-value | variance explained (r^2^) |
| --- | --- | --- | --- | --- | --- | --- | --- |
|  |  |  |  |  |  |  |  |
| departure date  (northbound) | (intercept) | 4.31 | 0.03 | 4.24/4.37 | 135.3 | <0.001 | 0.14 |
|  | temperature | 0.01 | 0.04 | -0.07/0.09 | 0.33 | 0.74 |  |
|  | precipitation | -0.05 | 0.03 | -0.12/0.03 | 1.26 | 0.21 |  |
|  | human density | 0.01 | 0.04 | -0.07/0.08 | 0.23 | 0.82 |  |
|  |  |  |  |  |  |  |  |
| arrival date  (northbound) | (intercept) | 4.75 | 0.02 | 4.7/4.8 | 198.61 | <0.001 | 0.38 |
|  | water availability | 0.07 | 0.04 | -0.01/0.15 | 1.63 | 0.1 |  |
|  | **temperature** | -0.13 | 0.05 | -0.22/-0.03 | 2.59 | **<0.01** |  |
|  | precipitation | 0.07 | 0.05 | -0.03/0.17 | 1.36 | 0.18 |  |
|  |  |  |  |  |  |  |  |
| departure date  (southbound) | (intercept) | 5.62 | 0.01 | 5.59/5.64 | 441.16 | <0.001 | 0.27 |
|  | NDVI | 0.01 | 0.01 | -0.02/0.04 | 0.5 | 0.62 |  |
|  | water availability | 0.02 | 0.01 | -0.01/0.04 | 1.23 | 0.22 |  |
|  | temperature | 0 | 0.01 | -0.03/0.02 | 0.3 | 0.77 |  |
|  | precipitation | -0.01 | 0.01 | -0.04/0.02 | 0.82 | 0.42 |  |
|  |  |  |  |  |  |  |  |
| arrival date  (southbound) | (intercept) | 0.01 | 0.01 | 5.7/5.75 | 430.61 | <0.001 | 0.06 |
|  | NDVI | 0.01 | 0.01 | -0.02/0.04 | 0.7 | 0.48 |  |
|  | temperature | 0.01 | 0.01 | -0.03/0.03 | 0.16 | 0.87 |  |
|  | precipitation | 0.01 | 0.01 | -0.03/0.02 | 0.4 | 0.69 |  |
|  |  |  |  |  |  |  |  |
| migration duration (northbound) | (intercept) | 3.69 | 0.04 | 3.61/3.77 | 87.89 | <0.001 | 0.55 |
|  | **water availability** | -0.18 | 0.05 | -0.29/-0.07 | 3.25 | **<0.01** |  |
|  | temperature | -0.04 | 0.05 | -0.15/0.07 | 0.76 | 0.45 |  |
|  | **precipitation** | 0.17 | 0.06 | 0.05/0.29 | 2.71 | **<0.01** |  |
|  | **human density** | -0.27 | 0.07 | -0.43/-0.12 | 3.42 | **<0.001** |  |
|  |  |  |  |  |  |  |  |
| migration duration (southbound) | (intercept) | 3.49 | 0.04 | 3.41/3.56 | 93.81 | <0.001 | 0.08 |
|  | **water availability** | 0.12 | 0.04 | 0.05/0.2 | 3.21 | **<0.01** |  |
|  | temperature | 0.03 | 0.04 | -0.05/0.12 | 0.8 | 0.43 |  |
|  | precipitation | 0.07 | 0.04 | -0.01/0.15 | 1.74 | 0.08 |  |
|  |  |  |  |  |  |  |  |
| number of stops  (northbound) | (intercept) | 0.09 | 0.1 | 1.69/2.09 | 18.71 | <0.001 | 0.41 |
|  | water availability | 0.12 | 0.13 | -0.35/0.15 | 0.75 | 0.46 |  |
|  | temperature | 0.12 | 0.13 | -0.39/0.1 | 1.16 | 0.25 |  |
|  | precipitation | 0.12 | 0.13 | -0.41/0.1 | 1.18 | 0.24 |  |
|  | human density | 0.12 | 0.13 | -0.48/0.04 | 1.64 | 0.1 |  |
|  |  |  |  |  |  |  |  |
| number of stops  (southbound) | (intercept) | 0.08 | 0.08 | 1.62/1.94 | 21.76 | <0.001 | 0.31 |
|  | water availability | 0.08 | 0.09 | -0.07/0.27 | 1.2 | 0.23 |  |
|  | temperature | 0.09 | 0.09 | -0.05/0.3 | 1.37 | 0.17 |  |
|  | precipitation | 0.09 | 0.1 | -0.06/0.31 | 1.35 | 0.18 |  |
|  |  |  |  |  |  |  |  |

Table A5. A summary table of model averaged estimates of GLMs examining the effects of principal components of temperature, precipitation, NDVI, water availability and human population density throughout annual cycle of WNC on the annual survival probability of adult and juvenile.

| dependent variables | explanatory variables | slope | std. error | 95% CI | *z* | p-value |
| --- | --- | --- | --- | --- | --- | --- |
|  |  |  |  |  |  |  |
| adult survival | (intercept) | 0.84 | 0.06 | 0.07/1.6 | 2.14 | 0 |
|  | comp. 1 | 0 | 0.02 | -0.31/0.31 | 0 | 1 |
|  | comp. 2 | 0 | 0.03 | -0.36/0.36 | 0.01 | 0.99 |
|  | comp. 3 | -0.03 | 0.03 | -0.45/0.39 | 0.13 | 0.9 |
|  | comp. 4 | 0 | 0.04 | -0.49/0.5 | 0.01 | 0.99 |
|  | comp. 5 | 0.02 | 0.04 | -0.54/0.58 | 0.06 | 0.95 |
|  |  |  |  |  |  |  |
| juvenile survival | (intercept) | 0.76 | 0.05 | 0.09/1.44 | 2.24 | 0 |
|  | comp. 1 | 0 | 0.02 | -0.27/0.28 | 0.03 | 0.97 |
|  | comp. 2 | -0.03 | 0.02 | -0.34/0.29 | 0.16 | 0.87 |
|  | comp. 3 | 0 | 0.03 | -0.37/0.37 | 0.01 | 0.99 |
|  | comp. 4 | 0.02 | 0.03 | -0.42/0.45 | 0.07 | 0.94 |
|  | comp. 5 | -0.05 | 0.04 | -0.54/0.44 | 0.2 | 0.84 |
|  |  |  |  |  |  |  |


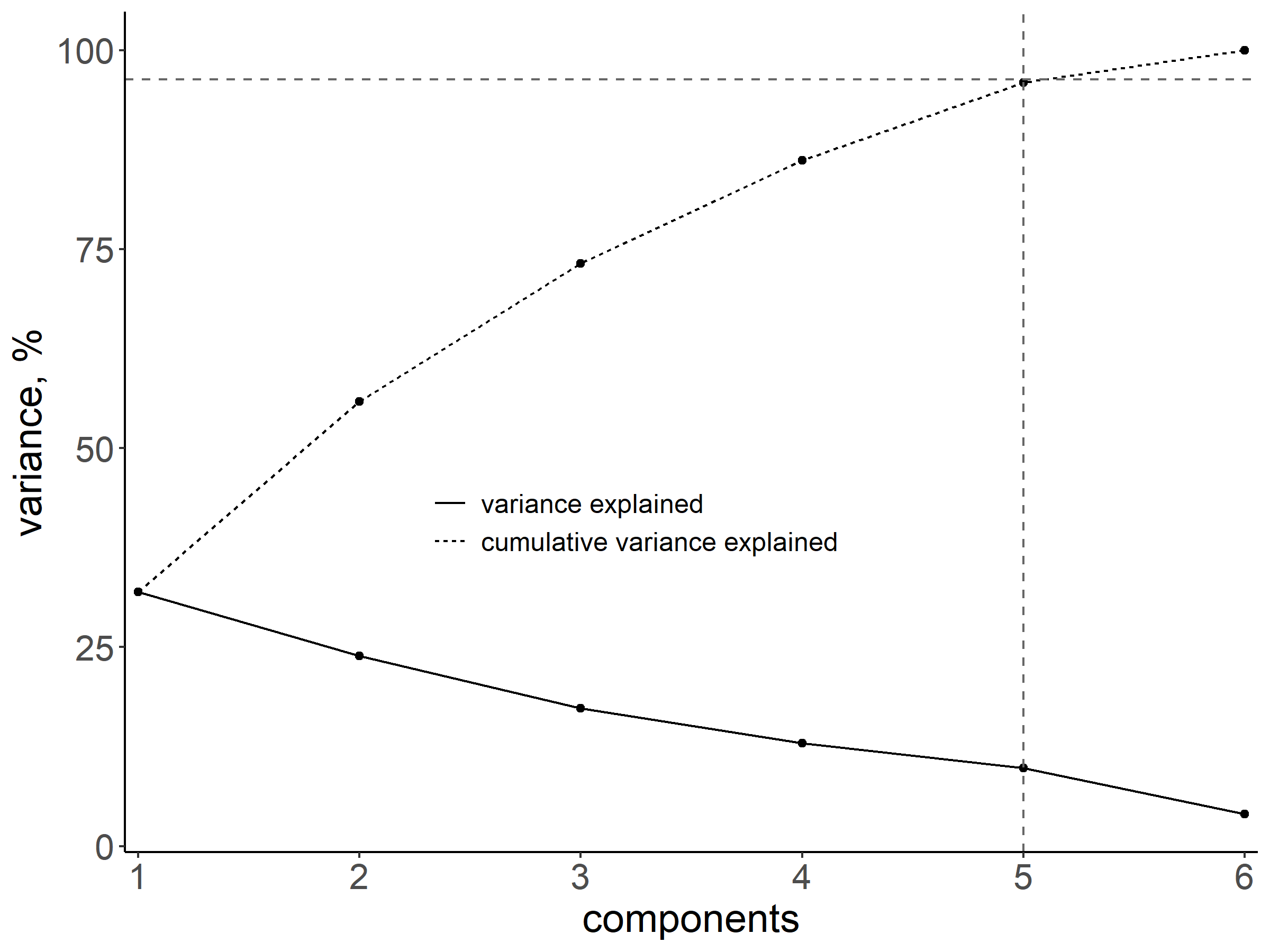


Fig. A1. A scree plot shows the variance explained by all six components (continuous line) as well as their cumulative variance (dotted line). The figure was produced using RStudio v2022.02.2 (<http://www.rstudio.com>) [[61](#_ENREF_61)].

Table A6. The coordinates of the principal components derived from temperature, precipitation, NDVI, water availability and human population density during relevant time periods at each of the staging sites visited by WNC.

| **variables** | **comp 1** | **comp 2** | **comp 3** | **comp 4** | **comp 5** | **comp 6** |
| --- | --- | --- | --- | --- | --- | --- |
| NDVI_Luan autumn | 0.85 | -0.31 | 0.11 | 0.18 | 0.22 | -0.28 |
| NDVI_Luan spring | -0.42 | 0.69 | 0.15 | -0.43 | 0.32 | 0.19 |
| NDVI_Mongolian summer | -0.79 | -0.25 | 0.52 | 0.16 | 0.14 | 0.05 |
| NDVI_Poyang winter | 0.50 | 0.51 | 0.61 | -0.26 | 0.01 | 0.22 |
| water_Luan autumn | 0.51 | 0.07 | -0.40 | -0.65 | -0.34 | -0.20 |
| water_Luan spring | -0.74 | 0.32 | 0.07 | -0.45 | 0.36 | 0.07 |
| water_Mongolian summer | -0.06 | -0.95 | 0.13 | 0.18 | 0.08 | 0.22 |
| water_Poyang winter | -0.71 | 0.17 | -0.49 | 0.24 | -0.35 | -0.21 |
| temperature_Luan autumn | 0.62 | -0.37 | 0.52 | 0.15 | 0.29 | -0.32 |
| temperature_Luan spring | 0.08 | 0.09 | -0.20 | 0.51 | 0.83 | 0.04 |
| temperature_Mongolian summer | 0.51 | 0.82 | -0.15 | -0.19 | 0.04 | 0.07 |
| temperature_Poyang winter | 0.82 | -0.17 | 0.50 | -0.10 | -0.19 | 0.10 |
| precipitation_Luan autumn | 0.46 | -0.56 | -0.50 | -0.09 | 0.04 | 0.47 |
| precipitation_Luan spring | 0.38 | 0.30 | 0.69 | 0.20 | -0.49 | 0.15 |
| precipitation_Mongolian summer | -0.90 | 0.03 | 0.43 | -0.02 | -0.03 | -0.11 |
| precipitation_Poyang winter | -0.38 | 0.14 | -0.02 | 0.74 | -0.51 | 0.17 |
| human_Luan | -0.44 | -0.24 | 0.81 | -0.26 | -0.06 | -0.12 |
| human_Mongol | -0.23 | -0.87 | 0.08 | -0.38 | -0.02 | 0.19 |
| human_Poyang | 0.32 | 0.73 | 0.21 | 0.54 | 0.14 | 0.12 |


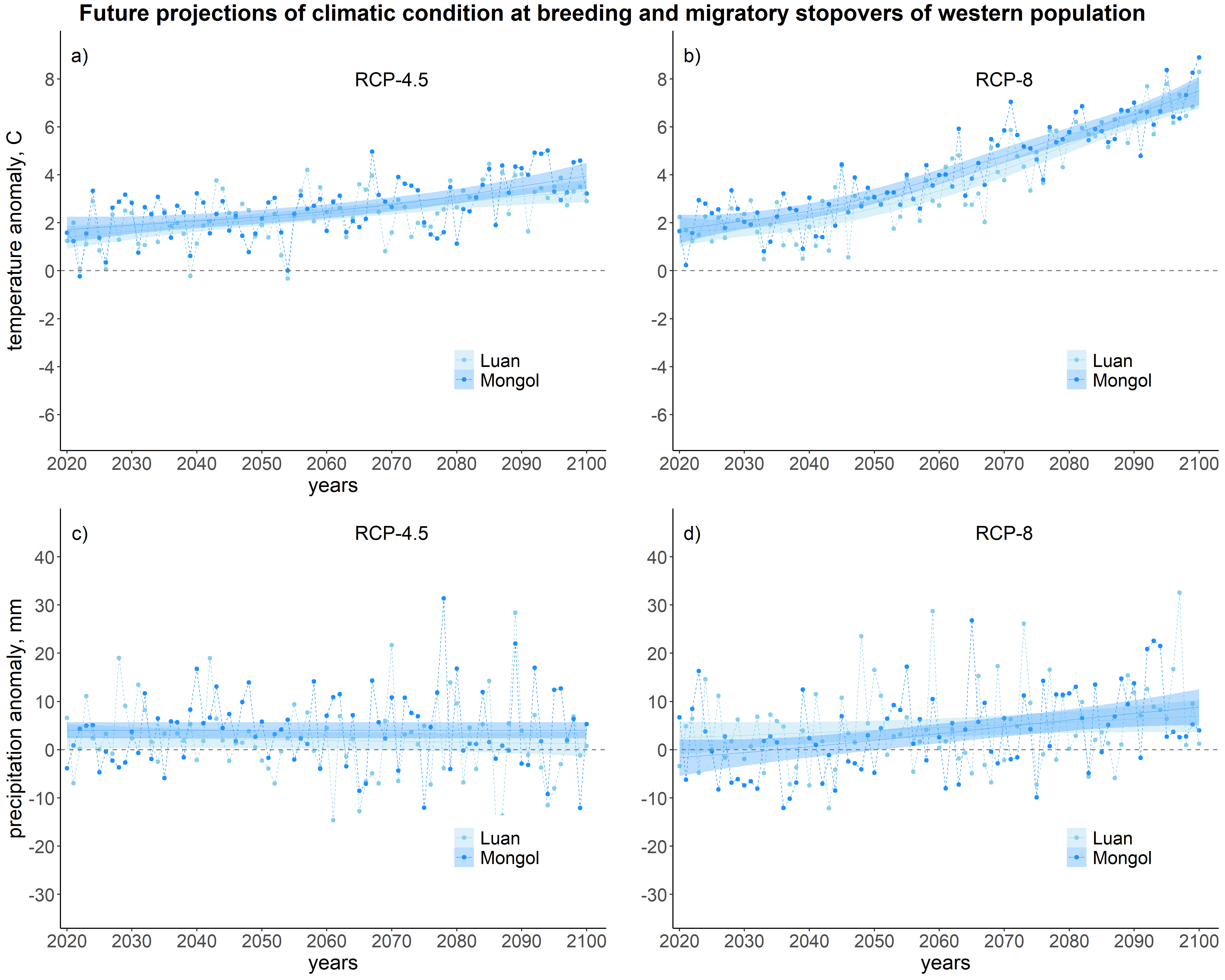


Fig. A2. Future trends of monthly mean temperature and precipitation in two climate change scenarios (Representative Concentration Pathway 4.5; Representative Concentration Pathway 8.5). Horizontal grey dashed lines indicate the average values between 1950-2019. The figure was produced using RStudio v2022.02.2 (<http://www.rstudio.com>) [[61](#_ENREF_61)].

Table A7. A summary table of GLMs examining the effects of laying date on the hatching success of WNC.

| dependent variables | explanatory variables | slope | std. error | 95% CI | *z* | p-value |
| --- | --- | --- | --- | --- | --- | --- |
|  |  |  |  |  |  |  |
| Hatching success | (intercept) | -1.47 | 0.98 | -3.4/0.44 | 0.13 | 0.13 |
|  | Laying date | 0.01 | 0.01 | -0.01/0.02 | 0.25 | 0.25 |
|  |  |  |  |  |  |  |


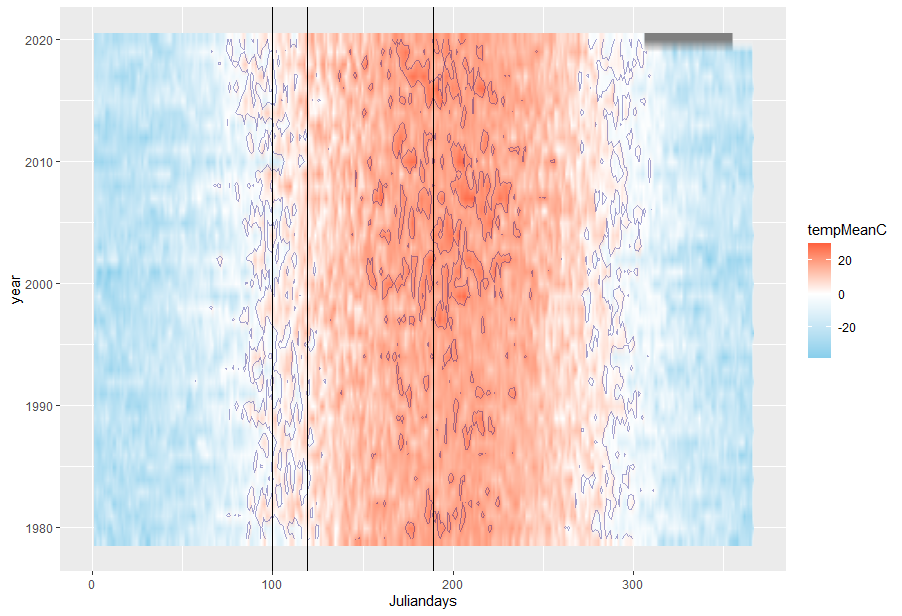


Fig. A3. Phenology of daily mean temperature over the past two decades in northeast Mongolia. The figure was produced using RStudio v2022.02.2 (<http://www.rstudio.com>) [[61](#_ENREF_61)].
